# F‐18 FDG PET‐CT for response evaluation in head and neck malignancy: Experience from a tertiary level hospital in south India

**DOI:** 10.1002/cnr2.1333

**Published:** 2021-03-03

**Authors:** Justin Benjamin, Julie Hephzibah, Nylla Shanthly, Regi Oommen, David Mathew, Simon Pavamani, Janakiraman Rajnikanth

**Affiliations:** ^1^ Department of Nuclear Medicine Christian Medical College Vellore India; ^2^ Department of Radiation Oncology Christian Medical College & Hospital Vellore India

**Keywords:** Deauville score, non‐responders, post‐RT PET‐CT, pre‐RT PET‐CT, responders

## Abstract

**Background:**

Head and neck squamous cell carcinoma (HNSCC) accounts for 90% of head and neck cancers. There has been no established qualitative system of interpretation for therapy response assessment using PET‐CT for HNSCC.

**Aim:**

To assess response evaluation of nodal status in post‐treatment PET‐CT scans in HNSCC using a 5‐point Likert scale (Deauville score [DS]).

**Methods and Results:**

Retro‐prospective analysis was performed of the nodal status of pre and post‐RT PET‐CT in patients diagnosed with HNSCC (n = 43) from May 2013 to March 2018. All eligible patients underwent a pre‐RT PET‐CT scan before the start of RT. Another post‐RT PET‐CT scan was performed 12 weeks after the completion of RT. The median time from completion of radiotherapy (RT) to post‐RT PET‐CT was 92 days; 80% of the patients had their post‐RT PET‐CT scan between 77 and 147 days after therapy. Of 43 patients (M/33, F/10, age range 18 to 80 years (median 54 years) selected for the study, good concordance was noted between DS and clinical response in these patients. The change in SUV from pre‐RT PET to post‐RT PET was analyzed using a paired *t*‐test. The *P*‐value was found to be statistically significant while comparing pre and post‐RT SUVmax levels showing that RT had significantly reduced the SUVmax levels of the nodes in DS 2‐3 groups whereas the number of patients was too small to allow a reliable calculation in DS 4‐5 groups. It was found that 36/39 patients with DS 1‐3 had no nodal recurrence showing a high NPV of 92.3%. Of the four patients with DS 4‐5, all had active disease showing PPV of 100%. Applying Fisher's exact test, the *P*‐value was found to be .004.

**Conclusion:**

DS seems to satisfy the requirements for a simple qualitative method of interpreting PET scans and for identifying patients requiring neck dissection. Consensus regarding qualitative assessment would facilitate standardization of PET reporting in clinical practice and enable comparative multicentric studies

## 1 INTRODUCTION

The annual incidence of head and neck squamous cell cancer (HNSCC) is about 680 000 new cases in the world, with a crude rate of 9.7 per 100 000 persons.^1^ In the management of HNSCC patients, functional imaging performed with 18F‐FDG PET‐computed tomography (PET‐CT) has several applications.^2^ F‐18 FDG PET‐CT is endorsed by the National Comprehensive Cancer Network (NCCN) guidelines for the diagnosis of occult primary and staging.^3^ PET‐CT is very accurate in detecting metastases or second primary tumors elsewhere in the body.^2^ Accurate delineation of target volumes is critical for intensity modulated radiation therapy (IMRT) treatments.

The role of imaging‐based biomarkers has been explored, but none of them can be used routinely to improve the selection of responders before the start of or during treatment.^4^, ^5^, ^6^, ^7^, ^8^

It has been well‐known that PET‐CT plays a significant role in the assessment of the response after chemoradiation (CRT) or radiation therapy (RT) alone. PET‐CT has shown a high negative predictive value (NPV) if performed at least 8 to 16 weeks after completion of treatment.^9^, ^10^

Early identification of poor responders or nonresponders may allow modification of the treatment plan (volume and doses) and/or implementation of alternative therapeutic strategies to intensify treatment. Few data are available on at least two PET‐CT scans over the whole RT course to evaluate changes in FDG uptake in the primary tumor as well as lymph node metastases.^11^, ^12^ The aim of this intensive monitoring during the treatment would also be to adjust the treatment plan according to the change in tumor volume in response to RT (adaptive RT).^13^

### 1.1 Aim

To assess response evaluation of nodal status in post treatment 18F‐FDG‐PET‐CT scans in HNSCC using a 5‐point Likert scale (Deauville score [DS]).

### 1.2 Objectives

1. To assign Likert scale (Deauville criteria score [DS]) and SUVmax to all follow up PET scans
2. To determine whether the interpretation of follow up PET scans can be improved
3. To categorize as responders vs non‐responders

## 2 MATERIALS AND METHODS

### 2.1 Methodology

The present study was retro‐prospective analysis of nodal status of pre‐ and post‐RT PET‐CT in patients diagnosed with HNSCC (n = 43) from May 2013‐March 2018.

Patients provided consent for the scans (but was under a waiver of informed consent approved for those in the retrospective series), and the study was approved by the Institutional Review Board.

### 2.2 Inclusion criteria

Patients with node positive (on pre‐RT PET scan) SCC of the larynx, hypopharynx and oropharynx, planned for organ preservation therapy with curative intent, and with no prior neck surgery, were included in the study. Patients were only eligible if the neck nodes demonstrated hypermetabolism on the pre‐RT PET scan.

### 2.3 Methods

All 43 eligible patients (33 male, 10 female; mean age ± SD, 53 ± 13 years) underwent a pre‐RT PET‐CT scan before the start of RT. Another post‐RT PET‐CT scan was performed 12 weeks after completion of RT. Patients without a pre‐RT PET‐CT study, without primary HNSCC, and with node negative scans were excluded.

The PET‐CT result helped in the decision regarding neck dissection versus observation. The multidisciplinary team ultimately decided whether patients with an equivocal post‐RT PET‐CT scan would undergo neck dissection or be scheduled for third PET (PET3) scan. The decision was based on various prognostic factors including initial N classification, human papillomavirus (HPV)/p16‐status if possible, performance status and also the post‐RT clinical response. Clinical follow‐up examinations were scheduled every 3 months during the first 2 years after either neck dissection or negative PET3, every 4 months in year 3 and every 6 months during the last 2 years of follow‐up.

PET imaging was carried out in accordance with our standard clinical PET protocol, the patients were injected intravenously with FDG 3.7 MBq/kg body weight to a maximum dose of 370 MBq after a 4 to 6 hour fasting period. All patients were imaged with an integrated PET‐CT system (Siemens Biograph True Point 6). After a 45 minutes—1‐hour uptake period at rest, images were acquired for 2 minutes per bed position. At baseline and for follow‐up studies, the CT scan was acquired together with the PET scan. CT scans helped in attenuation correction and anatomical localization.

All PET scans were visually evaluated by Nuclear Medicine Physicians regarding metabolic response.^14^

In this study two experienced readers, without prior knowledge of the clinical outcome, re‐evaluated all patients regarding metabolic neck node response. Images were assessed and SUVmax levels obtained using Multimodality workplace (Siemens Syngo 2009B, VE36 A SL10P25 sMMWP SPO4). Metabolic responses were scored according to the Deauville score.^15^

Overall assessment is denoted by the overall score, which is the highest score among the scores for all the neck nodes. The Deauville scores are given in Table 1 together with the categories used in the present study.

**TABLE 1:** Deauville criteria regarding neck node response to treatment

Deauville score	FDG uptake
1	No uptake
2	Uptake ≤ mediastinum
3	Uptake > mediastinum ≤ liver
4	Uptake moderately increased compared with liver at any site
5	Uptake markedly increased compared with liver at any site and/or new sites of disease

**FIGURE 1:**
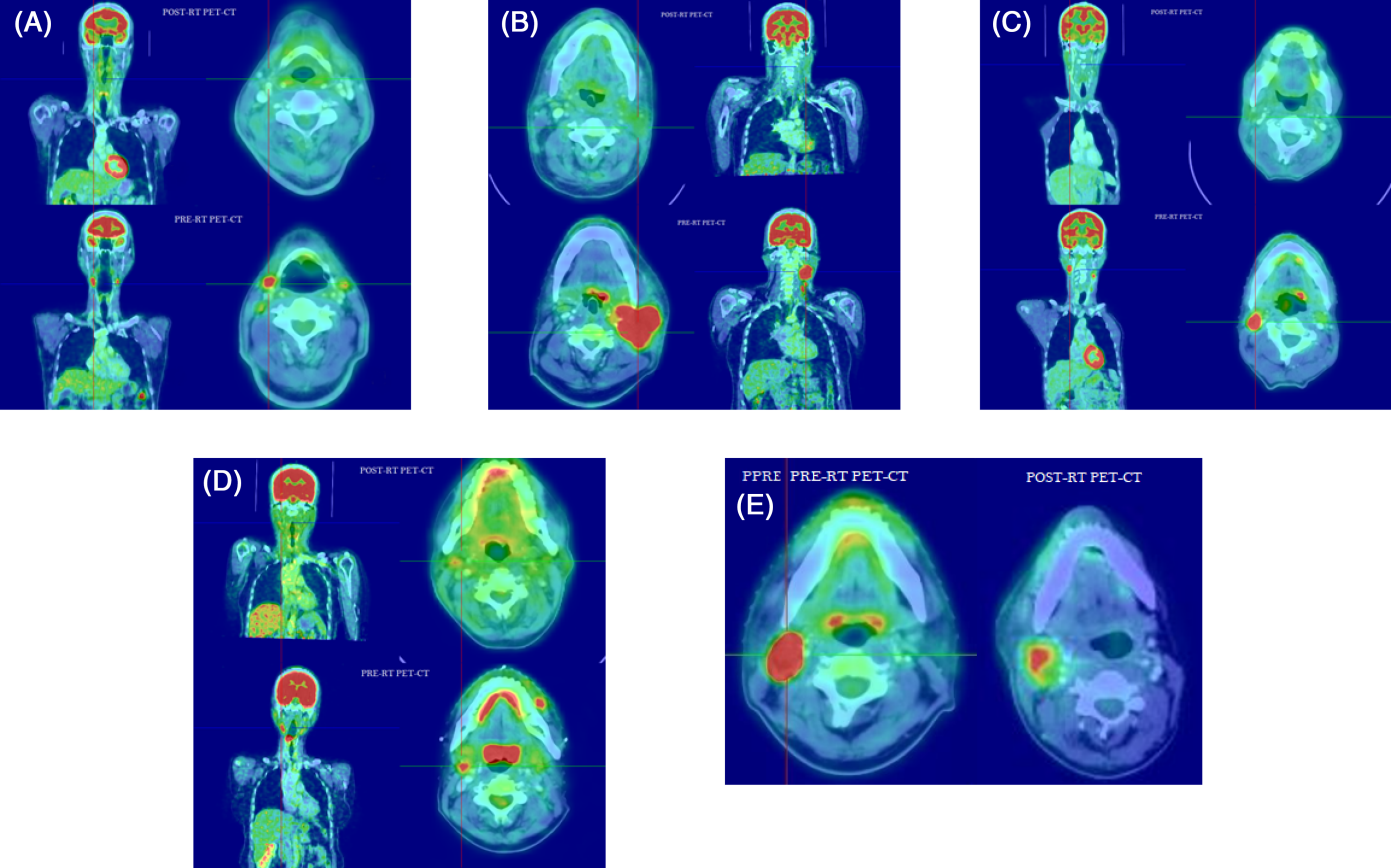
Pre‐ and post‐RT PET‐CT images of patients with DS 1‐5. A, 65/M with Carcinoma tongue, T4N2M0 with pre‐RT PET showing bilateral level 2a with highest SUV of 7.85 and post RT PET after 12 weeks showing no nodal recurrence—DS 1. B, 50/M with Carcinoma left oropharynx, T1N2bM0 with pre‐RT PET showing left level 2, 3 nodes with highest SUV of 9.12 and post‐ RT PET after 12 weeks showing significant regression. Post‐RT PET showed left level 3 node with SUV of 2.97 and when compared with mediastinal blood pool was found to have DS 2. C, 48/M with Carcinoma supraglottis, T3N2cM0 with pre‐RT PET showing right level 2a, 3 nodes with highest SUV of 21.45 and post‐ RT PET after 16 weeks showing significant regression. Post‐RT PET showed right level 2 node with SUV of 2.47 and when compared with mediastinal blood pool was found to have DS 3. D, 55/F with carcinoma right pyriform sinus, T3N0M0 with pre‐RT PET showing right level 2, 3 nodes with highest SUV of 9.68 and post‐ RT PET showing residual right level 3 node. Post‐RT PET showed right level 3 node with SUV of 5.63 and was higher than mediastinal blood pool and liver and was found to have DS 4. E, 51/M with carcinoma hypopharynx, T4N2aM0 with pre‐RT PET showing right level 2, 3 nodes with highest SUV of 26.78. Post‐RT PET showed right level 2, 3 nodes with SUV of 15.27 and was found to have DS 5

Examples of corresponding PET images are shown in the following figures (Figure 1A–E). If FDG uptake was seen in the neck nodes, the highest uptake was scored.

### 2.4 Definition of response assessment

A complete response to RT in the neck and regional control (RC) was defined as no residual or recurrent tumor in the neck after completion of RT until the last date of follow‐up in our institution. A residual tumor in the neck was categorized as persistent tumor, according to the pathology report, after neck dissection planned as a result of the post‐RT PET or PET3 scan. The Deauville criteria Likert scale was dichotomized into responders (scores 2 and 3) and nonresponders (scores 4 and 5).

A post‐RT PET scan demonstrating a DS of 2 or 3 was considered true negative if the patient did not have any tumor on histopathological correlation after neck dissection, or any neck relapse during follow‐up. In false‐negative post‐RT PET scans, either residual tumor was found or a relapse occurred during the follow‐up period. Scans reported as showing DS of 4 or 5 were considered true‐positive if neck dissection revealed residual tumor, according to the pathology report, or if there was progressive neck disease in patients with non‐operable disease during the clinical follow‐up. It would be considered false‐positive on post‐RT PET scans, if no residual tumor, according to the pathology report, was found in the neck dissection specimen.

### 2.5 Statistical analysis

Data were summarized using the mean (SD)/Median for continuous variables based on the normality. The categorical data were expressed as number and frequency. The change in SUV from pre‐RT PET to post‐RT PET was analysed using paired *t* test. The association between the categorical data were analysed using Fisher's exact test. The log‐rank test was used to compare the categorical predictors over the recurrence. A *P* value of <.05 was considered statistically significant. All the analysis were performed using STATA I/c 15 software.

## 3 RESULTS

### 3.1 Patient and tumor characteristics

**FIGURE 2:**
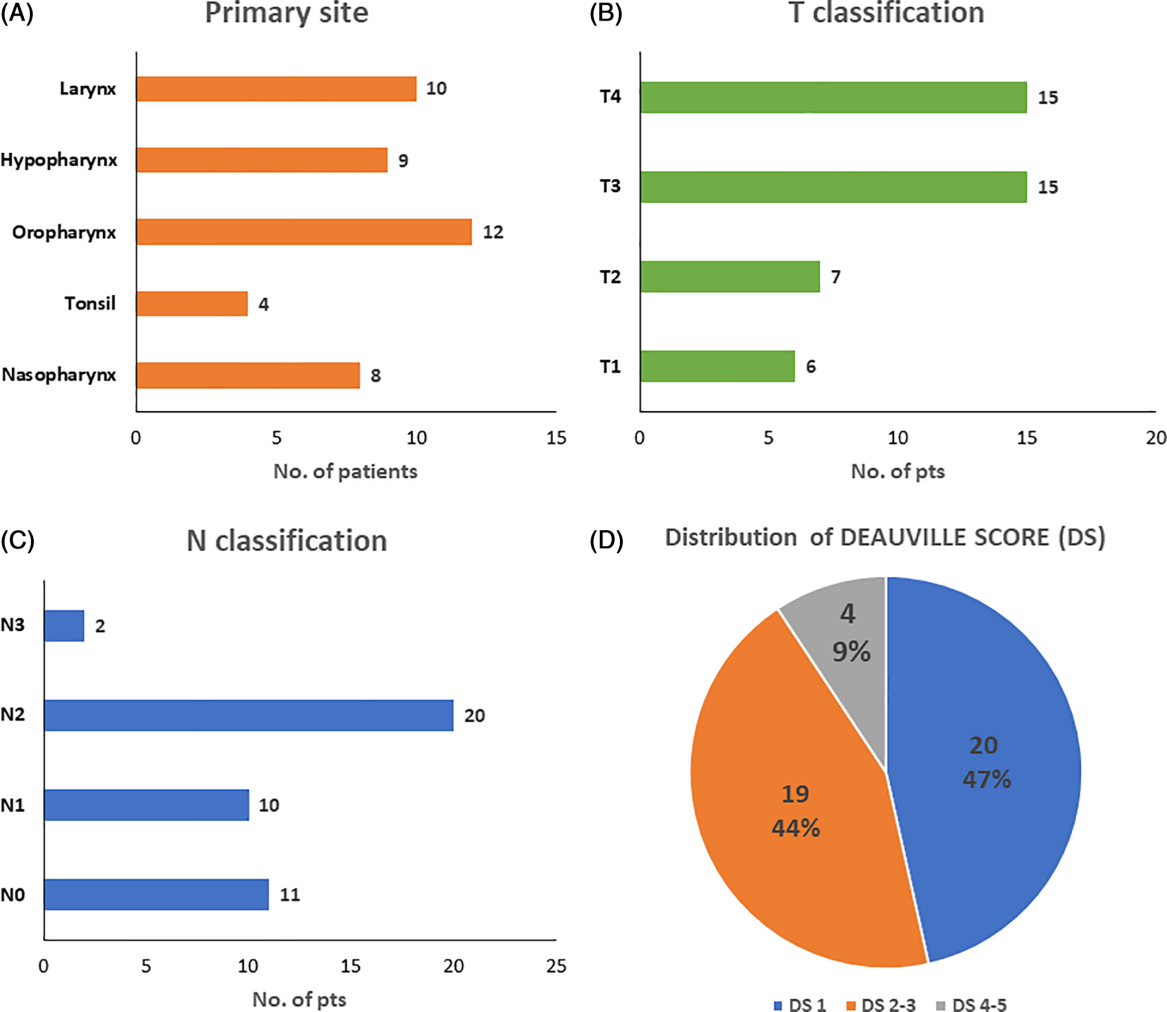
Distribution of primary site, T staging, N staging and Deauville score. A, primary site; B, T staging; C, N staging; D, distribution of Deauville score

Forty‐three patients (33 male and 10 female) who fulfilled the criteria were included in the given study period (Table 2). The primary site of tumor was classified as nasopharynx (18.60%), tonsil (9.3%), oropharynx (27.9%), hypopharynx (20.93%) and larynx (23.25%; Figure 2A).

**TABLE 2:** Patient characteristics

Characteristic	Value	Characteristic	Value
Age (years), median (range)	18‐80, 54	T classification (n = 43)	
Gender		T1	6 (13.9%)
Female	10 (23.3%)	T2	7 (16.3%)
Male	33 (76.7%)	T3	15 (34.9%)
Primary site (n = 43)		T4	15 (34.9%)
Nasopharynx	8 (18.6%)	N classification	
Tonsil	4 (9.3%)	N0	11 (25.6%)
Oropharynx (other than above)	12 (27.9%)	N1	10 (23.2%)
Hypopharynx	9 (20.93%)	N2	20 (46.5%)
Larynx	10 (23.25%)	N3	2 (4.7%)
Treatment			
Radiotherapy	43		
Concurrent chemotherapy	41		

The median time from completion of RT to post‐RT PET‐CT was 92 days; 80% of the patients had their post‐RT PET‐CT scan between 77 and 147 days after therapy.

### 3.2 TNM classification

There were six patients in the T1 group, seven patients in T2 group and 15 each in T3 and T4 groups, respectively (Figure 2B,C). There were 11 patients in N0 group, 10 patients in N1 group, 20 patients in N2 group and remaining 2 of them were in N3 group. No one had distant metastases.

### 3.3 Deauville criteria score

Deauville score was assessed based on the nodal FDG activity of post RT‐PET scans when compared with pre‐RT‐PET scans. It was compared with mediastinal, liver blood pool or both. Then it was categorized into the following groups: DS 1, DS 2‐3 and DS 4‐5. DS 1was found in 20 patients, DS 2‐3 in 19 patients and DS 4‐5 in 4 patients (Figure 2D).

Of the 20 patients in the DS‐1 group, the SUVmax levels of the most active neck node in pre‐RT PET scans were ranging from 2.6 to 23.72 (median SUVmax 8.8) and the neck nodes did not show any FDG activity in their respective post‐RT PET scans.

**FIGURE 3:**
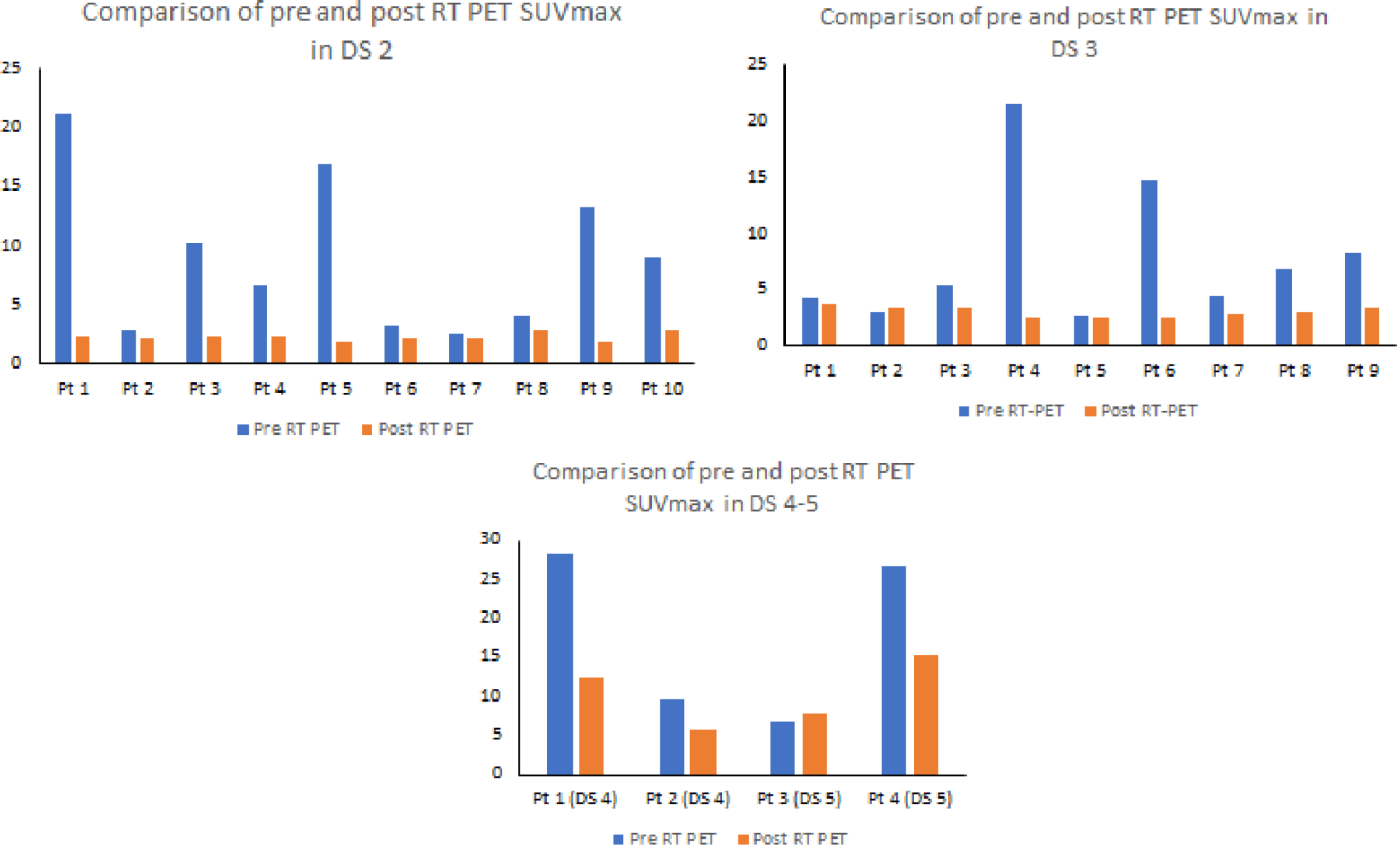
Panel of comparisons between SUV's of Pre‐RT and post‐RT PET in DS 2‐5 groups

Of the 10 patients in the DS‐2 group, SUVmax levels of the most active neck node in pre‐RT PET scans of DS‐2 group were ranging from 2.5 to 21.1 (median SUVmax 7.9) and as compared to SUVmax levels measured in their respective post‐RT PET scans were ranging from 1.83 to 2.97 (median SUVmax 2.27). Using Wilcoxon signed‐rank test, the *P*‐value was found to be .0051 whereas comparing pre and post‐RT SUVmax levels showing that RT/CRT had significantly reduced the SUVmax levels of the nodes (Figure 3).

Of the nine patients in the DS‐3 group, SUVmax levels of the most active neck node in pre‐RT PET scans were ranging from 2.7 to 21.45 (median SUVmax 5.3) and as compared to SUVmax levels measured in their respective post‐RT PET scans were ranging from 2.42 to 3.65 (median SUVmax 3.06). Three out of these nine patients had histopathologically confirmed residual tumor cells on follow‐up. Using Wilcoxon signed‐rank test, the *P*‐value was found to be .01 whereas comparing pre and post‐RT SUVmax levels showing that RT/CRT had significantly reduced the SUVmax levels of the nodes in this group (Figure 3).

Of the 4 patients in the DS 4‐5 group, SUVmax levels of the most active neck node in pre‐RT PET scans were ranging from 6.69 to 28.26 (median SUVmax 18.23) and as compared to SUVmax levels measured in their respective post‐RT PET scans were ranging from 5.63 to 15.27 (median SUVmax 10.13) and all of them had disease progression. The number of patients was too small to allow a reliable calculation for value for comparing the effect of RT on pre‐RT and post‐RT SUVmax levels (Figure 3).

### 3.4 Follow‐up

The median follow‐up time from the date of completion of RT was 12 months (range 4‐53 months) and two patients died within the period of the study, one of them was due to recurrent cholecystitis and the other due to disease progression. Of the 43 patients, seven were found to have disease progression during the follow‐up period from the date of the scan to the last patient encounter at our institution. Of these, progression was confirmed in 5 (71.4%) patients by tissue diagnosis and 2 (38.6%) patients by imaging and clinical follow‐up. They were all in DS 3‐5 groups (Table 3).

**TABLE 3:** Follow up in DS groups 2–5

	DS 2	DS 3	DS 4–5
No nodal recurrence	10	6	0
Disease progression	0	3	4
Total	10	9	4

There was no disease progression in DS 1‐2 groups.

It was found that 36/39 patients with DS 1‐3 had no nodal recurrence showing a high NPV of 92.3%. Of the four patients with DS 4‐5 all had active disease showing PPV of 100%.

Applying Fisher's exact test, the *P*‐value was found to be .004. This highlighted that DS 2‐3 was useful in predicting the absence of nodal recurrence and DS 4‐5 in predicting disease progression.

## 4 DISCUSSION

It was imperative that there was development of functional imaging like F‐18 FDG PET‐CT because it can identify an occult primary tumor and is very accurate in detecting metastases or second primary tumors elsewhere in the body, and precise delineation of target volumes is critical for IMRT treatments. The pre‐eminence of functional over conventional imaging in response evaluation consists of its ability to semiquantitatively assess glucose uptake as a cancer cell viability indicator.

The response of HNSCC to treatment depends on various factors such as clinical history (previous treatments), tumor characteristics (stage and biology), surrounding microenvironment and host immunoresponse.

After concurrent CRT for locally advanced HNSCC, locoregional recurrence develops in 20% to 30% of patients, typically in the gross tumor volume, suggesting that dose escalation or additional interventions could help in improving local tumor control. PET‐CT might be used to identify the biological target volume (BTV) inside the target volume. Evidence exists that local recurrences characteristically occur within areas of high FDG uptake.^16^ Such areas could be treated with a boost dose using IMRT techniques to reduce the risk of recurrence. In recent years, there has been increasing interest in the role of PET‐CT acquired during CRT with the aim to identify tumor responsiveness at an early stage of treatment.^17^

Early identification of poor responders or nonresponders may allow modification of the treatment plan (volume and doses) and/or implementation of alternative therapeutic strategies to intensify treatment. Therefore, utilising a Likert scale such as DS which could categorize the responders from non‐responders, would prevent unnecessary prophylactic neck dissections.

In our study, there was a good concordance between the DS and SUVmax. All methods of assessment predicted RC with high significance and almost similar to each other. The DS also showed encouraging results in discriminating responders from nonresponders on PET scans judged as equivocal. There was significant difference in SUVmax between patients with and without residual/recurrent tumor after completion of RT in DS 2‐3 groups only.

Nevertheless, measurement of SUVmax is affected by technical, biological and physical factors,^18^ and in spite of an attempt to establish common criteria there are still many diverse ways of calculating and presenting SUVmax. Published cut‐off values are usually specific to the method and to the institution.^19^ In this study, as also shown previously, SUVmax did not give any additional value when compared with visual inspection in the clinical setting.^20^, ^21^

Treatment response is an important factor for planning management and determining prognosis in HNSCC. It has been established that PET‐CT has great potential to predict treatment response and helps in the early detection of residual or recurrent disease, which allows salvage therapy to be implemented and helps in predicting complete response, avoiding the need for unnecessary intervention.^22^, ^23^ Known limitations also include low PPVs, ascribed to inflammation and post‐treatment effects, such as edema, fibrosis, asymmetry, and anatomic distortion. The high NPVs observed in these studies indicate that a negative post‐treatment scan is suggestive of absence of active disease, thereby influencing treatment planning.^24^

There is so far a need for consensus on qualitative assessment and reporting of PET scan results. There has been no established interpretation system described in the literature to help readers classify the post‐treatment PET‐CT findings in a reliable manner in patients with HNSCC. There have been multicentre trials in Hodgkin lymphoma where the DS has been validated and approved.^15^, ^25^, ^26^ In 48 patients with HNSCC using routine clinical follow‐up as the reference, Krabbe et al. used a five‐point scale in a serial PET evaluation, 3, 6, 9 and 12 months after treatment and demonstrated an overall PPV of 51% and an NPV of 100%.^27^ Marcus et al. in 2014 introduced and validated the Hopkins criteria, a five‐point Likert scale which is very similar to DS.^28^

In a prospective PET study done by Porceddu et al. qualitative interpretation on focal uptake was assessed in relation to uptake in adjacent tissue and the liver.^29^ There were three different categories: “positive” (for residual tumor), “negative” and “equivocal.” NPV was found to be as high as 97.1% in the long‐term follow‐up even though all recurrences, irrespective of when they occurred, were included in the “false‐negative” group.^12^ As mentioned above, the NPV of DS used in this study was 92.3%. This may be due to the fact that the studies were scheduled 12 weeks after RT. Higher accuracy of PET scans has been noted in recent studies and meta‐analyses, where it was scheduled later than 7 weeks after treatment.^30^, ^31^ The treatment regimens were not uniform. In our setting, single modality treatment with RT was rarely used and CRT was used in >95% of the patients which aligned with similar studies in which CRT is more frequent. In this study, the PET results were characterized as false‐negative if recurrent cancer was found at any stage during the follow‐up period in contrast to the studies by Krabbe et al and Marcus et al in which a 6‐month limit for false‐negative scans was applied.^28^, ^32^

Post‐RT PET‐CT scan with DS 1 was defined as “no FDG uptake,” which is a category of limited clinical value. A likely cause of no FDG uptake could be complete necrosis of the neck node.

The benefit of adding DS to the PET report is apparent as responses can be categorized, and are distinct and easily interpreted by the oncologist or head and neck surgeon who have to act upon the PET result. In the present study, 44.18% (19 of 43 post‐RT PET‐CT scans) were in DS 2‐3 group. The percentage of equivocal scans is higher than that found in previous studies.^33^ By considering the equivocal PET scans together with those scored as 2 or 3 (responders), we were able to correctly categorize 16 of the 19 patients. In three remaining patients, categorized in DS 2‐3 group, we diagnosed recurrences greater than 5 months after therapy completion. The group of PET scans assessed as equivocal was small and statistics should be interpreted with caution.

However, DS adequately categorized 84.2% of the equivocal PET scans, which is encouraging. In the group of PET scans judged as equivocal, SUVmax provided no additional predictive value.

In this study, we chose to focus on the neck nodes with the highest FDG uptake in relation to treatment response. We did not evaluate the primary site response but it would be of interest to investigate whether using DS could minimize the number of equivocal scans here as well.^34^

The study results need to be interpreted within the context of this study. HPV status was not available for all the patients in the study, especially earlier in the study period.

## 5 CONCLUSION

Equivocal PET scan in HNSCC poses clinical dilemma. DS based on Likert scale for assessment of FDG metabolism in neck nodes following organ preservation therapy in HNSCC is a promising tool to overcome this problem. All patients with DS 4 or 5 on post‐treatment PET scan can be considered as non‐responders and should be routinely scheduled for neck dissection.

Deauville score seems to satisfy the requirements for a simple qualitative method of interpreting PET scans and for identifying patients requiring neck dissection. Consensus regarding qualitative assessment would facilitate standardization of PET reporting in clinical practice and enable comparative multicentric studies.

## CONFLICT OF INTEREST

The authors declare there is no conflict of interest.

## ETHICAL STATEMENT

Patients provided consent for the scans (but was under a waiver of informed consent approved for those in the retrospective series), and the study was approved by the Institutional Review Board.

## AUTHOR CONTRIBUTIONS

**Justin Benjamin:** Conceptualization; data curation; formal analysis; funding acquisition; investigation; methodology; resources; software; supervision; writing‐original draft; writing‐review and editing. **Regi Oommen:** Conceptualization; formal analysis; investigation; methodology; project administration; resources; supervision; validation; writing‐review and editing. **Julie Hephzibah:** Conceptualization; formal analysis; methodology; project administration; resources; validation; visualization; writing‐review and editing. **David Mathew:** Conceptualization; formal analysis; methodology; supervision; validation; visualization; writing‐review and editing. **Nylla Shanthly:** Formal analysis; methodology; writing‐review and editing. **Simon Pavamani:** Conceptualization; funding acquisition; project administration; supervision. **janakiraman rajinikanth:** Conceptualization; methodology; project administration; supervision; validation.

## Data Availability

The data that support the findings of this study are available from the corresponding author, [RO], upon reasonable request.
